# Supplementary material for: Cold-induced nucleosome dynamics linked to silencing of Arabidopsis FLC
Source: Nat Commun. 2025 Jul 1;16:5550. doi: 10.1038/s41467-025-60735-z (PMC12216206; doi:10.1038/s41467-025-60735-z)
Supplement: Supplementary file 1 — Supplementary Information [file 41467_2025_60735_MOESM1_ESM.pdf]

Supplemental Figure 1

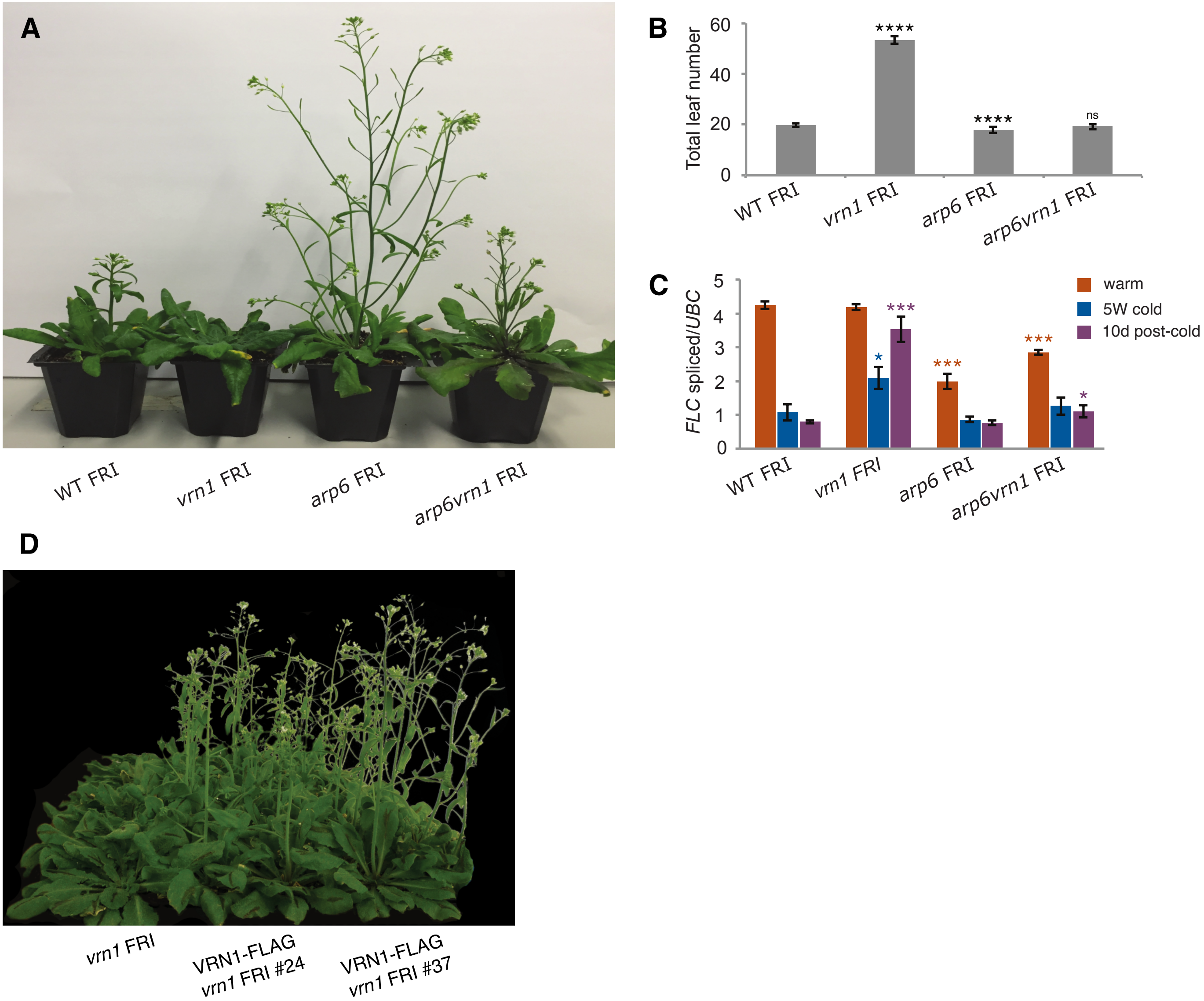

SuppFig1 – VRN1 is involved in chromatin regulation.
(A) Flowering phenotype of plants grown at warm temperature for 30 days after 5 weeks of cold. (B) Flowering time assessed by total leaf number. Bars and error bars show the mean +/- SD from twenty-four individual plants for each genotype. (C) *FLC* expression normalized to *UBC21* (AT5G25760) in WT FRI, *vrn1-4* FRI, *arp6-1* FRI, and *arp6-1 vrn1-4* FRI seedlings grown at warm temperature, subjected to 5 weeks of cold (5W cold), and 10 days back to warm temperature after 5 weeks of cold (10d post-cold). Bars and error bars show the mean +/- SD. (B, C) ns *p*-value > 0.05, **p*-value < 0.05, ****p*-value < 0.001 , *****p*-value < 0.0001 from two-tailed Student's *t*-test for differences in each mutant relative to WT FRI. (D) Flowering phenotype of *vrn1-4* FRI and VRN1-FLAG *vrn1-4* FRI transgenic lines #24 and #37.

Supplemental Figure 2

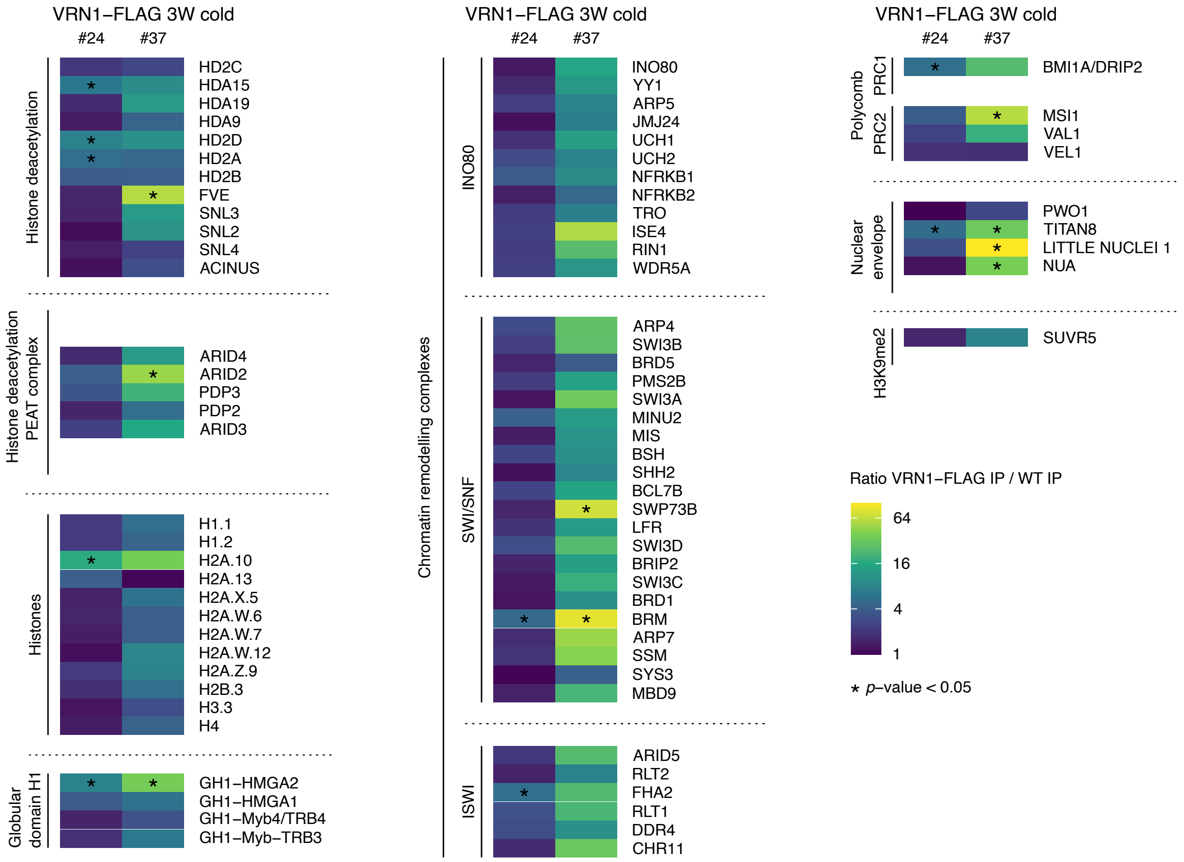

SuppFig2 – VRN1 IP-MS in plants in the cold.
Proteins enriched in VRN1-FLAG IP relative to WT IP from plants subjected to 3 weeks of cold (3W cold). The colour scale indicates the enrichment levels in a logarithmic scale. *p-value < 0.05 from two-tailed Student's t-test.

Supplemental Figure 3

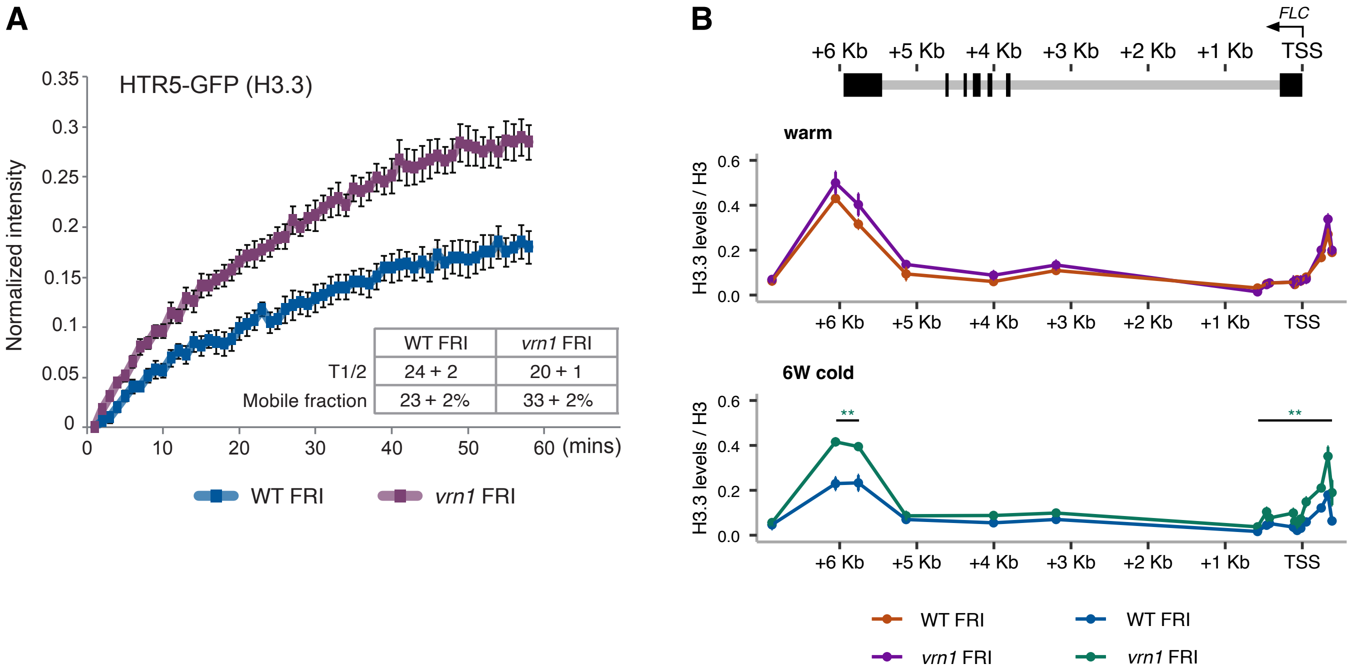

SuppFig3 – VRN1 is involved in chromatin regulation.
(A) FRAP normalized fluorescence intensity for pHTR5:HTR5::GFP (H3.3) in WT FRI and *vrn1-4* FRI backgrounds. Points and error bars show the mean +/- SD, n=10. The average half-time recovery (T1/2) and percentage of mobile fraction for each genotype are shown. (B) HTR5::GFP (H3.3) ChIP-qPCR levels as percent of input normalized to H3 in WT FRI and *vrn1-4* FRI plants grown at warm temperature or subjected to 6 (6W) weeks of cold. Points and error bars show the mean +/- SD, n=2. Statistical differences were assessed over the two peaks at the 5' and 3' ends and the region in between. ***p*-value < 0.01, from two-tailed Student's *t*-test. Schematic representation of the *FLC* locus on the top of the plot with exons as black boxes and introns as grey lines. An arrow at the TSS indicates the direction of transcription.

Supplemental Figure 4

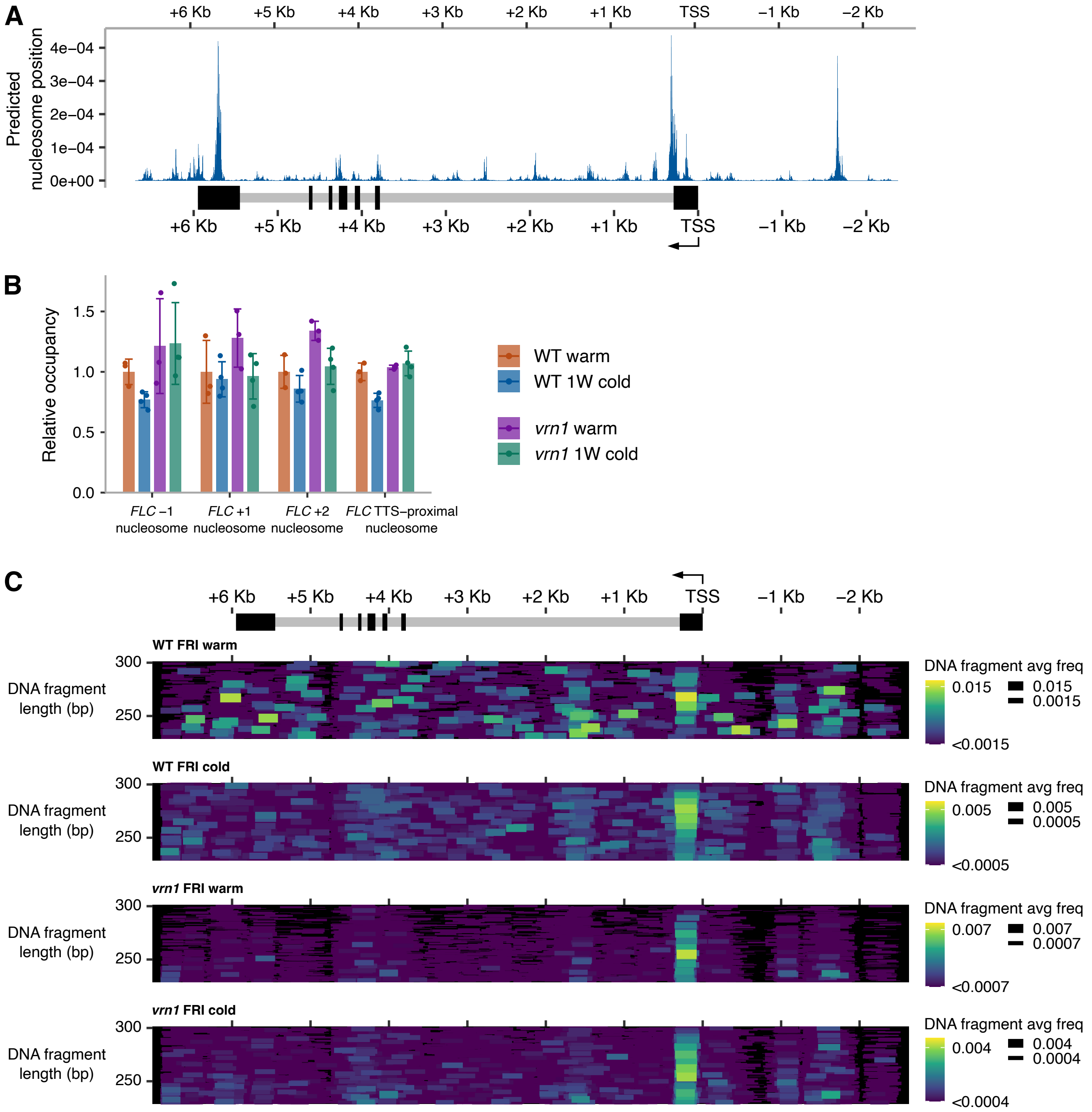

SuppFig4 – Nucleosome position and occupancy at *FLC*.
(A) Nucleosome position at *FLC* predicted *in silico* based on genomic DNA sequence[85]. Schematic representation of the *FLC* locus on the bottom of the plot with exons as black boxes and introns as grey lines. (B) Relative nucleosome occupancy levels from MNase-seq for WT FRI and *vrn1-4* FRI at warm temperature or after 1 week of cold (1W cold) normalized to *GAPDH* (AT1G42970) and *ACT7* (AT5G09810; see Methods). Bars and error bars show the mean +/- SD. Points show individual biological replicates. (C) MNase-protected fragments between 230 bp and 300 bp reconstituted from paired reads plotted as coloured horizontal segments. The genomic position on the *x*-axis is aligned and in scale with the schematic representation of the *FLC* gene on the top; exons in black, introns in grey. The *y*-axis shows the DNA length. Both segment colour and width scales represent the average frequency of a detected DNA fragment within the *FLC* locus in WT FRI and *vrn1-4* FRI at warm temperature and or 1 week of cold (cold).

Supplemental Figure 5

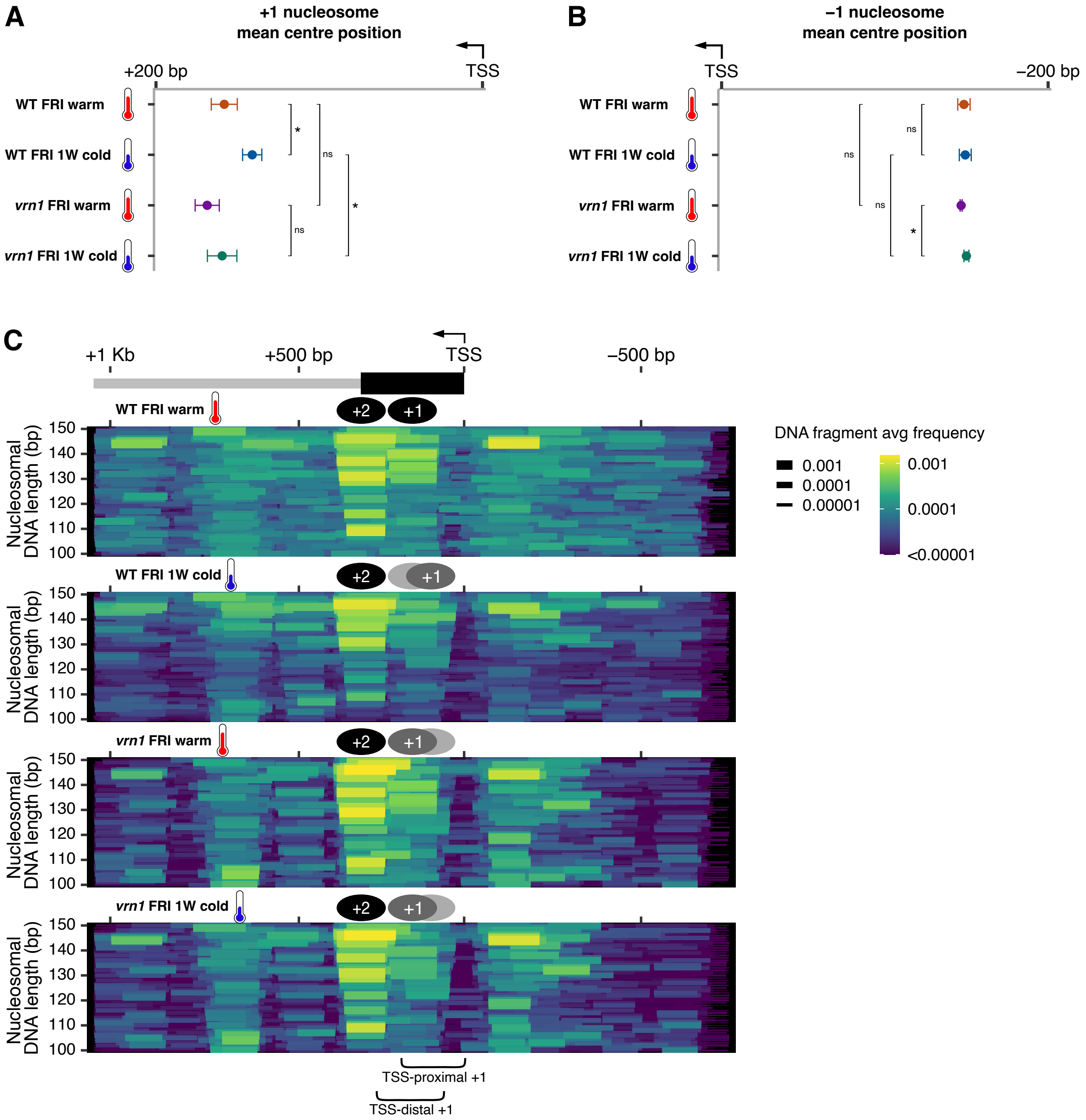

SuppFig5 – Repositioning of the +1 nucleosome at *FLC*.
(A,B) Quantification of the mean centre position of nucleosomal fragments reconstituted from paired MNase-seq reads within the 200 bp downstream *FLC* TSS (A) or upstream *FLC* TSS (B). Points and error bars show the mean +/- SD. ns p-value >= 0.05, *p-value < 0.05 from two-tailed Student's *t*-test. (C) MNase-seq for the 5' end of *FLC* in WT FRI and *vrn1-4* FRI seedlings at warm temperature or after 1 week of cold (1W cold). The genomic position on the *x*-axis is aligned and in scale with the schematic representation of the *FLC* on the top with exon 1 in black and intron 1 in grey. The nucleosomal DNA reconstituted from paired reads were plotted as coloured horizontal segments.The *y*-axis resolves the nucleosomal DNA by fragment length. Both segment colour and width scales show the average frequency of a detected DNA fragment. A representation of the +1 and +2 nucleosome positions infered from the data were added as schematics above each plot. The windows used to calculate the ratio of +1 TSS-proximal to TSS-distal in Fig3E are shown in the bottom.

Supplemental Figure 6

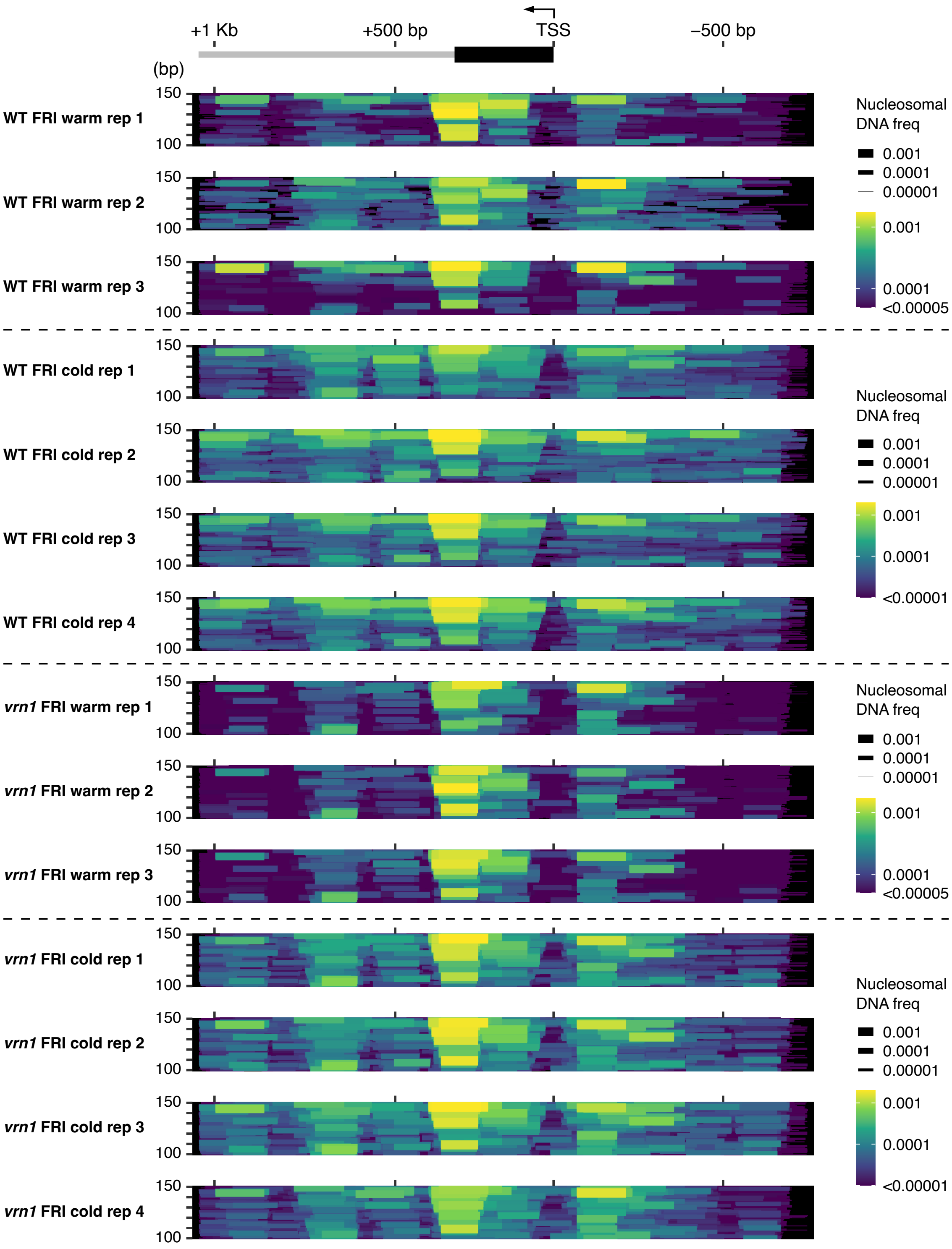

SuppFig6 – MNase-seq profiles at the 5' end of *FLC*.
MNase-seq plots for the 5' end of the *FLC* for each replicate separately. Three replicates for WT FRI and *vrn1-4* FRI at warm temperature and four replicates for WT FRI and *vrn1-4* FRI after 1 week of cold (cold). The genomic position on the *x*-axis is aligned and in scale with the schematic representation of the 5' end region of *FLC*; exon 1 in black, intron 1 in grey. The *y*-axis shows the nucleosomal DNA length. Nucleosomal DNA fragments were plotted as coloured horizontal segments. Colour and width scales show the frequency of each nucleosomal DNA fragment.

Supplemental Figure 7

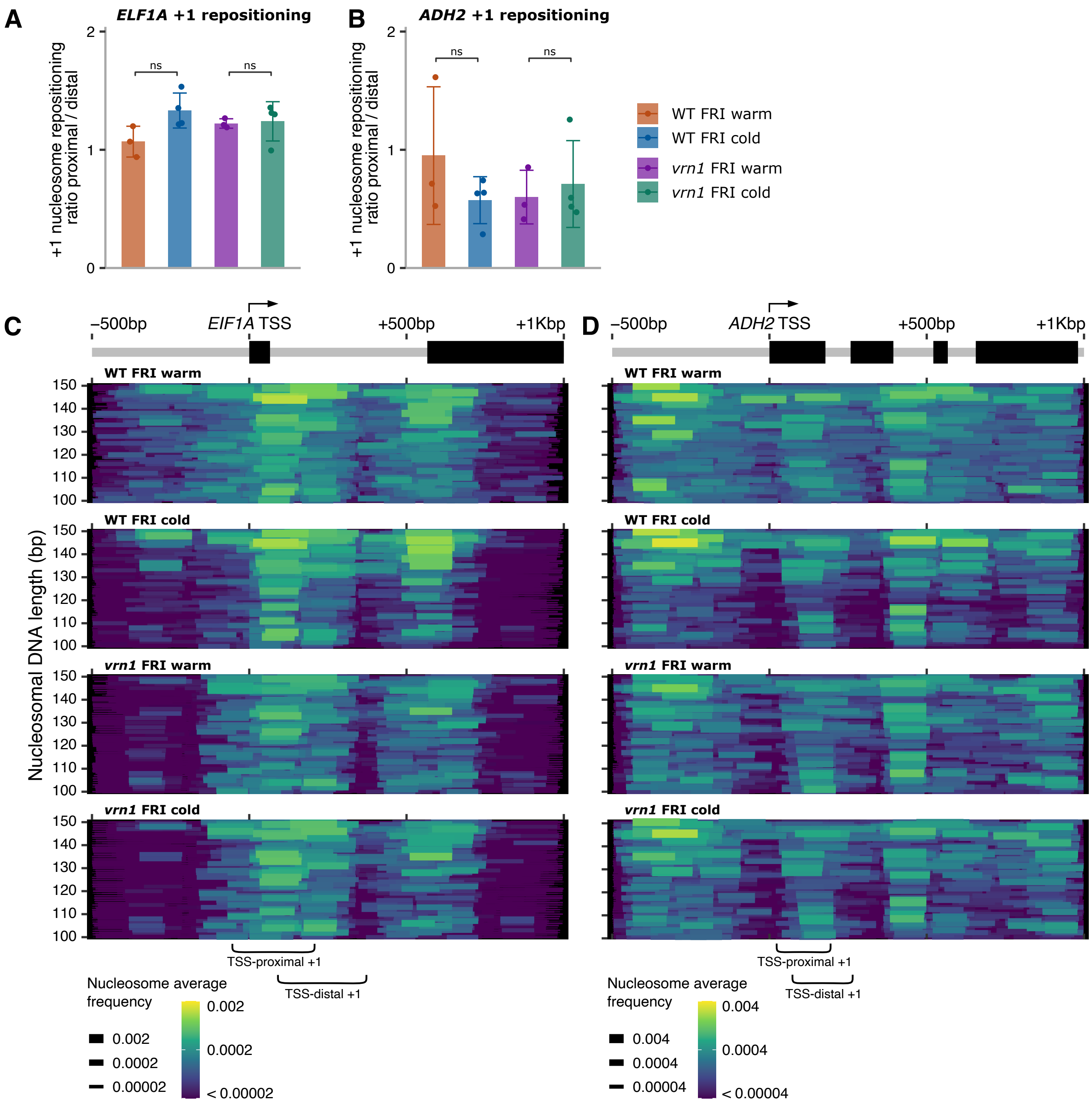

SuppFig7 – Repositioning of the +1 nucleosome at other genes.
(A-D) MNase-seq in WT FRI and *vrn1-4* FRI plants grown at warm temperature or after 1 week of cold (cold). +1 nucleosome repositioning plots showing the ratio of TSS-proximal nucleosomal fragments to TSS-distal at the (A) *EIF1A* (AT5G60390) and (B) *ADH2* (AT5G43940) loci. (A, B) Bars and error bars represent the mean +/- SD. Points represent individual biological replicates. ns indicates *p*-value >= 0.05, **p*-value < 0.05 from two-tailed Student's *t*-test. (C, D) MNase-seq plots for the 5' end of the *EIF1A* and *ADH2* loci. The *x*-axis shows the region from 500 bp upstream to 1 Kbp downstream of the TSS. The *y*-axis shows the nucleosomal DNA length. Colour and width scales show the average frequency of a nucleosomal DNA fragment. Schematics of the gene on the top with exons in black. The brackets on the bottom indicate the windows used to quantify +1 nucleosome repositioning shown on panels A and B.

Supplemental Figure 8

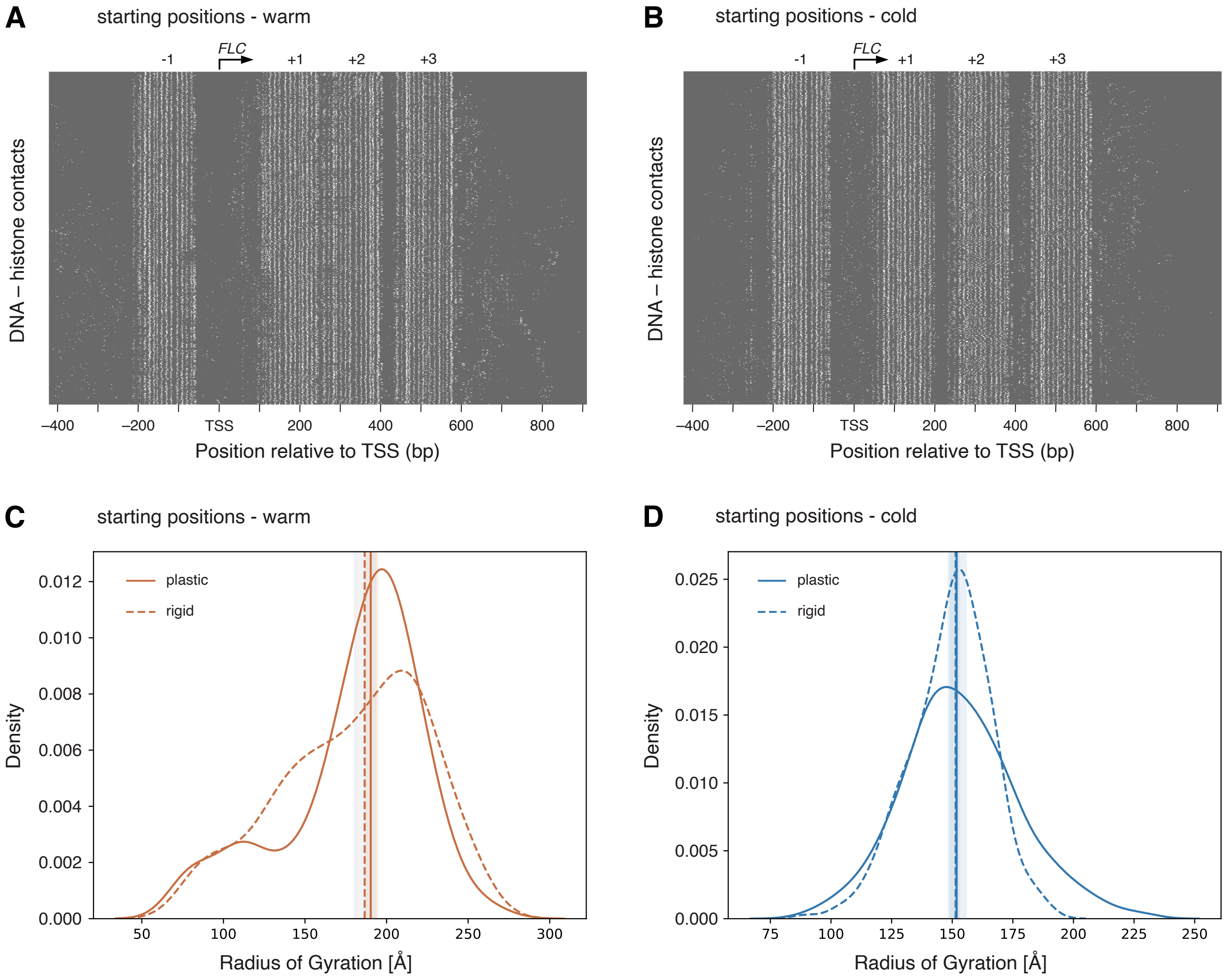

SuppFig8 – Simulations of *FLC* chromatin with warm vs cold positioning.
(A, B) Contact maps for the simulations of chromatin at the 5' end of *FLC* at physiological salt with warm-positioned (A) and cold-positioned (B) nucleosomes. Each line along the *y*-axis is a simulation snapshot, with white dots indicating the presence of a histone-DNA contact at a given basepair on the *x*-axis. (C, D) Distribution of the radii of gyration for the simulations at physiological salt of plastic chromatin (solid line), in which nucleosome breathing and sliding motions were allowed, or rigid chromatin (dashed line), in which nucleosome breathing and sliding was constrained; with nucleosomes with warm positioning (C) or cold positioning (D). Vertical lines represent the median, and shaded area represents the confidence interval (95%).

Supplemental Figure 9

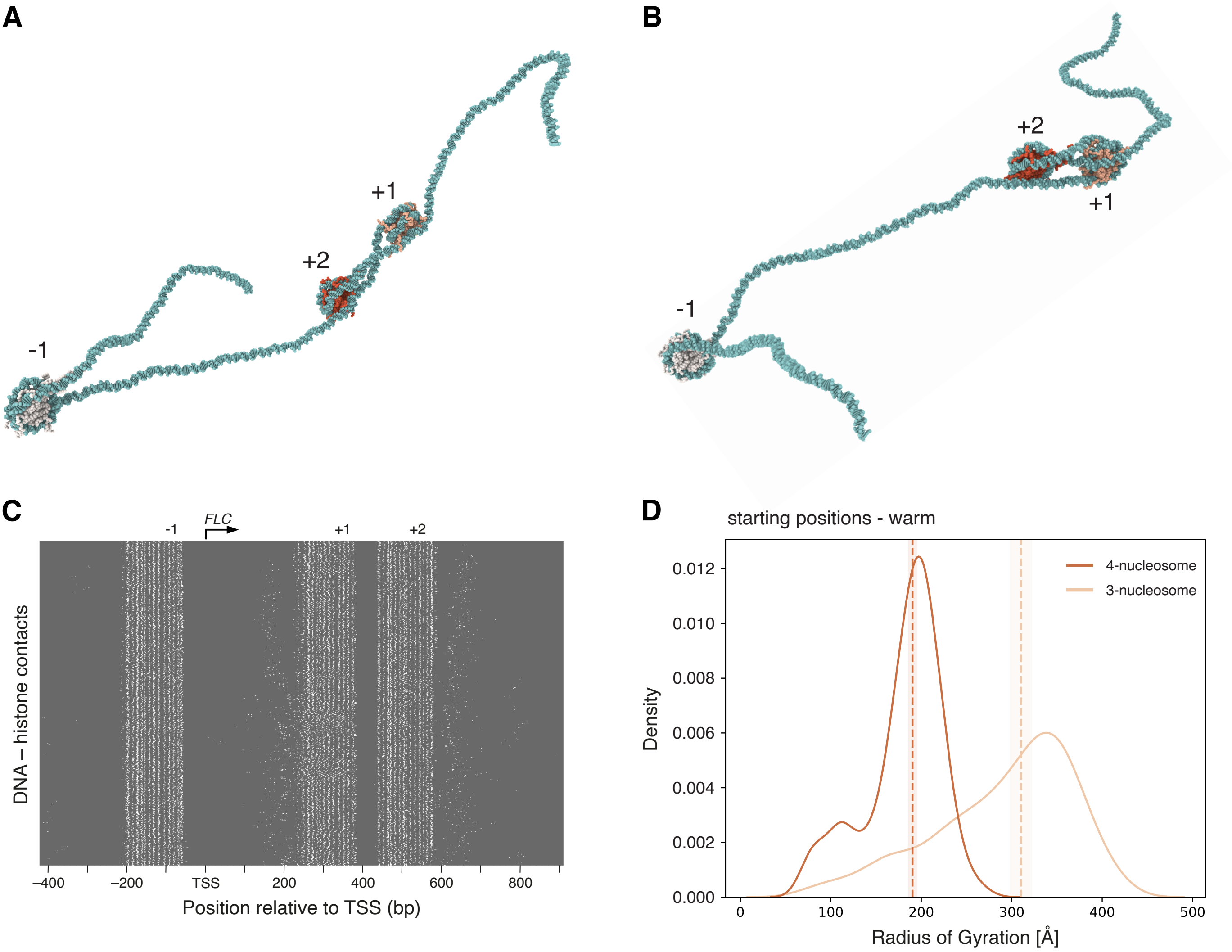

SuppFig9 – Simulations of an alternative (3-nucleosome) chromatin structure at the 5' end of *FLC*.
(A,B) Two representative snapshots from the simulations of an alternative *FLC* chromatin scenario where the presumed +1 and +2 nucleosome positions detected *in vivo* would derive from one nucleosome with two alternative positions. (C) Contact maps for those simulations. Each line along the *y*-axis is a simulation snapshot, with white dots indicating the presence of a histone-DNA contact at a given basepair on the *x*-axis. (D) Distribution of the radii of gyration for the alternative simulations (light coloured line) vs the simulations with 4 nucleosomes with warm positioning (**Fig5A,C**; dark coloured line). Vertical dashed lines represent the median, and shaded area represents the confidence interval (95%).

Supplemental Figure 10

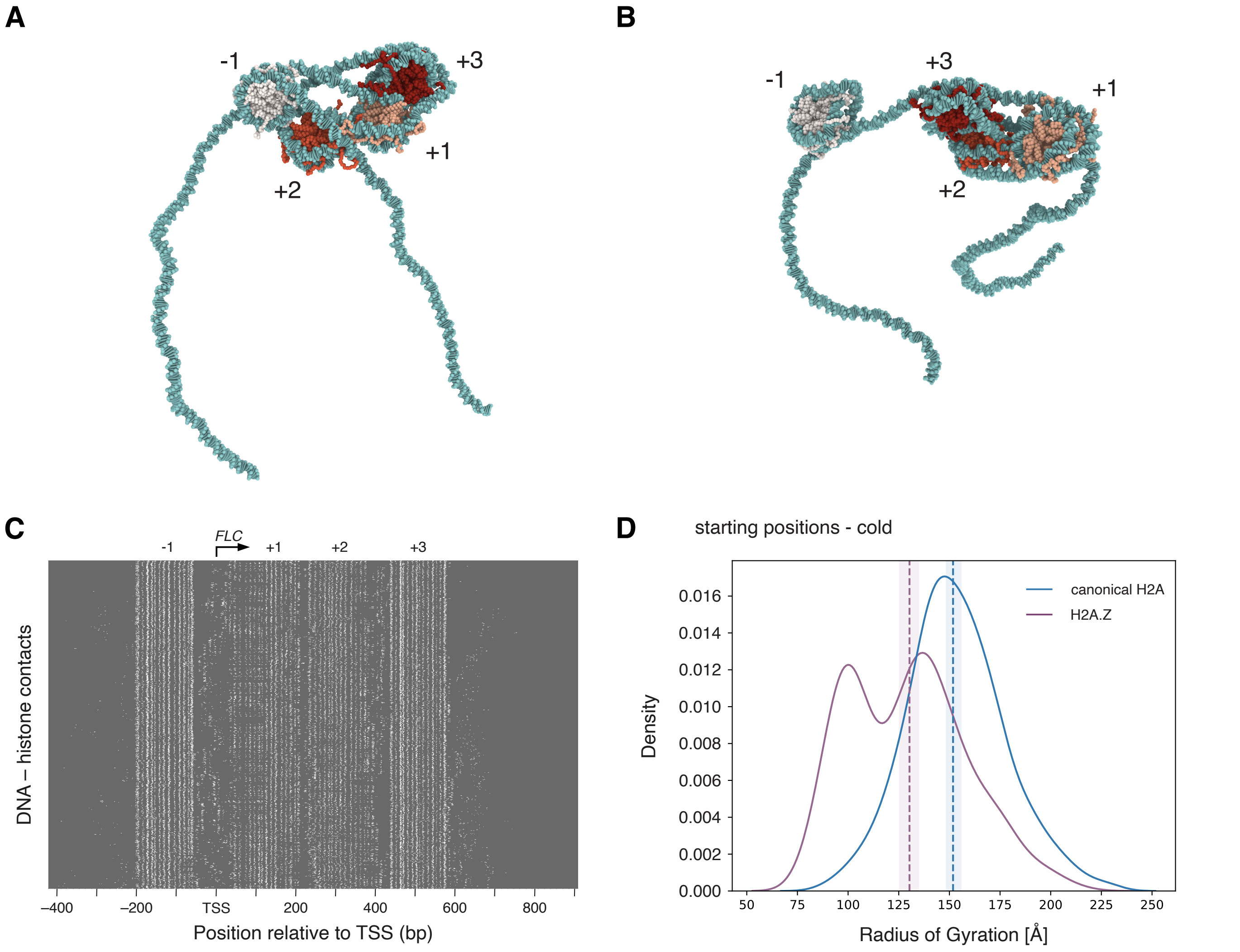

SuppFig10 – Simulations of *FLC* chromatin with H2A.Z-containing nucleosomes.
(A, B) Representative snapshots from the simulations of *FLC* chromatin at physiological salt with nucleosomes with cold positioning, containing the histone variant H2A.Z at the nucleosomes +1 and +2. (C) Contact maps for H2A.Z-containing *FLC* chromatin with cold-positioned nucleosomes. Each line along the *y*-axis is a simulation snapshot, with white dots indicating the presence of a histone-DNA contact at a given basepair on the *x*-axis. (D) Distribution of the radii of gyration for the simulations of *FLC* chromatin with cold-positioned nucleosomes containing canonical histones (**Fig5B,C**; blue line) or the histone variant H2A.Z at the +1 and +2 nucleosomes (purple line). Vertical lines represent the median, and shaded area represents the confidence interval (95%).
