# Supplementary material for: Cold-induced nucleosome dynamics linked to silencing of Arabidopsis FLC
Source: Nat Commun. 2025 Jul 1;16:5550. doi: 10.1038/s41467-025-60735-z (PMC12216206; doi:10.1038/s41467-025-60735-z)
Supplement: Supplementary file 2 — Description of Additional Supplementary Files [file 41467_2025_60735_MOESM2_ESM.pdf]

Description of Additional Supplementary Files

Supplementary Dataset 1
Description: List of all primers.
Supplementary Dataset 2
Description: VRN1-FLAG IP-MS in plants in the warm.
Supplementary Dataset 3 –
Description: VRN1-FLAG IP-MS in plants in the cold.
Supplementary Dataset4
Description: List of all bait sequences used for library enrichment.
