# Supplementary material for: Cold-induced nucleosome dynamics linked to silencing of Arabidopsis FLC
Source: Nat Commun. 2025 Jul 1;16:5550. doi: 10.1038/s41467-025-60735-z (PMC12216206; doi:10.1038/s41467-025-60735-z)
Supplement: Supplementary file 7 — Reporting Summary [file 41467_2025_60735_MOESM7_ESM.pdf]

nature portfolio

Corresponding author(s): Caroline Dean

Last updated by author(s): May 7, 2025

Reporting Summary

Nature Portfolio wishes to improve the reproducibility of the work that we publish. This form provides structure for consistency and transparency in reporting. For further information on Nature Portfolio policies, see our Editorial Policies and the Editorial Policy Checklist.

Statistics

For all statistical analyses, confirm that the following items are present in the figure legend, table legend, main text, or Methods section.

n/a	Confirmed	
☐	☒	The exact sample size (*n*) for each experimental group/condition, given as a discrete number and unit of measurement
☒	☐	A statement on whether measurements were taken from distinct samples or whether the same sample was measured repeatedly
☐	☒	The statistical test(s) used AND whether they are one- or two-sided<br>*Only common tests should be described solely by name; describe more complex techniques in the Methods section.*
☒	☐	A description of all covariates tested
☒	☐	A description of any assumptions or corrections, such as tests of normality and adjustment for multiple comparisons
☐	☒	A full description of the statistical parameters including central tendency (e.g. means) or other basic estimates (e.g. regression coefficient) AND variation (e.g. standard deviation) or associated estimates of uncertainty (e.g. confidence intervals)
☐	☒	For null hypothesis testing, the test statistic (e.g. *F*, *t*, *r*) with confidence intervals, effect sizes, degrees of freedom and *P* value noted<br>*Give P values as exact values whenever suitable.*
☒	☐	For Bayesian analysis, information on the choice of priors and Markov chain Monte Carlo settings
☒	☐	For hierarchical and complex designs, identification of the appropriate level for tests and full reporting of outcomes
☒	☐	Estimates of effect sizes (e.g. Cohen's *d*, Pearson's *r*), indicating how they were calculated

Our web collection on statistics for biologists contains articles on many of the points above.

Software and code

Policy information about availability of computer code

Data collection	No software was used.
Data analysis	Proteome Discoverer 3.0 was used for IP-MS data analysis. LAMMPS (version 3rd March 2020), Visual Molecular Dynamics (VMD). and Open Visualization Tool (OVITO) software were used for MD simulations data analises and image rendering.All the codes for MNase-seq data processing, analysis, and plotting are available on GitHub (https://github.com/Miguel-Montez/Targeted-MNase-seq-analysis).

For manuscripts utilizing custom algorithms or software that are central to the research but not yet described in published literature, software must be made available to editors and reviewers. We strongly encourage code deposition in a community repository (e.g. GitHub). See the Nature Portfolio guidelines for submitting code & software for further information.

Data

Policy information about availability of data

All manuscripts must include a data availability statement. This statement should provide the following information, where applicable:

- Accession codes, unique identifiers, or web links for publicly available datasets
- A description of any restrictions on data availability
- For clinical datasets or third party data, please ensure that the statement adheres to our policy

All the codes for MNase-seq data processing, analysis, and plotting are available on GitHub (https://github.com/Miguel-Montez/Targeted-MNase-seq-analysis). The MNase-seq data generated in this study have been deposited at the NCBI Gene Expression Omnibus (GEO) under the accession GSE274066. The mass spectrometry

proteomics data have been deposited to the ProteomeXchange Consortium via the PRIDE partner repository with the dataset identifiers PXD054597 (VRN1-FLAG IP-MS in the warm) and PXD060769 (VRN1-FLAG IP-MS during cold).

Research involving human participants, their data, or biological material

Policy information about studies with human participants or human data. See also policy information about sex, gender (identity/presentation), and sexual orientation and race, ethnicity and racism.

Reporting on sex and gender	This information has not been collected.
Reporting on race, ethnicity, or other socially relevant groupings	This information has not been collected.
Population characteristics	This information has not been collected.
Recruitment	No recruitment involved.
Ethics oversight	Not part of this study.

Note that full information on the approval of the study protocol must also be provided in the manuscript.

Field-specific reporting

Please select the one below that is the best fit for your research. If you are not sure, read the appropriate sections before making your selection.

☒ Life sciences ☐ Behavioural & social sciences ☐ Ecological, evolutionary & environmental sciences

For a reference copy of the document with all sections, see nature.com/documents/nr-reporting-summary-flat.pdf

Life sciences study design

All studies must disclose on these points even when the disclosure is negative.

Sample size	Sample sizes were not predetermined.
Data exclusions	No data was excluded.
Replication	Experiments were repeated at least once to confirm reproducibility.
Randomization	Samples were allocated into experimental groups randomly.
Blinding	Investigators were not blinded.

Reporting for specific materials, systems and methods

We require information from authors about some types of materials, experimental systems and methods used in many studies. Here, indicate whether each material, system or method listed is relevant to your study. If you are not sure if a list item applies to your research, read the appropriate section before selecting a response.

Materials & experimental systems

n/a	Involved in the study	
☐	☒	Antibodies
☒	☐	Eukaryotic cell lines
☒	☐	Palaeontology and archaeology
☒	☐	Animals and other organisms
☒	☐	Clinical data
☒	☐	Dual use research of concern
☐	☒	Plants

Methods

n/a	Involved in the study	
☒	☐	ChIP-seq
☒	☐	Flow cytometry
☒	☐	MRI-based neuroimaging

Antibodies

Antibodies used	Antibodies used in this study: Anti-GFP (Abcam, ab290), anti-FLAG antibody (Sigma, F1804), anti-H3 (Abcam, ab1791), anti-H3K27me3 (Millipore, 07-449), H2AK119ub (Cell Signalling Technology, #8240), anti-H2B (Abcam, ab1790).

Validation

All these antibodies have been validated by the commercial providers and in multiple published research works.

Dual use research of concern

Policy information about dual use research of concern

Hazards

Could the accidental, deliberate or reckless misuse of agents or technologies generated in the work, or the application of information presented in the manuscript, pose a threat to:

No	Yes	
☒	☐	Public health
☒	☐	National security
☒	☐	Crops and/or livestock
☒	☐	Ecosystems
☒	☐	Any other significant area

Experiments of concern

Does the work involve any of these experiments of concern:

No	Yes	
☒	☐	Demonstrate how to render a vaccine ineffective
☒	☐	Confer resistance to therapeutically useful antibiotics or antiviral agents
☒	☐	Enhance the virulence of a pathogen or render a nonpathogen virulent
☒	☐	Increase transmissibility of a pathogen
☒	☐	Alter the host range of a pathogen
☒	☐	Enable evasion of diagnostic/detection modalities
☒	☐	Enable the weaponization of a biological agent or toxin
☒	☐	Any other potentially harmful combination of experiments and agents

Plants

Seed stocks

All genotypes used in this study have Columbia (Col-0) background with a functional FRIGIDA allele introgressed from the ecotype San Feliu-2 (Lee & Amasino, 1995). vin3-4 (SALK_004766.51.00.x 77), arp6-1 (Garlic_599_G0 31), pHTA9::HTA9-GFP (Kumar & Wigge, 2010), pHTR5:HTR5::GFP (Wollmann et al., 2012), and vrn1-4 FRI mutant obtained from a fast-neutron mutagenesis (Sung & Amasino, 2004).

Novel plant genotypes

VRN1-FLAG C-terminal fusion was generated by cloning a genomic VRN1 (AT3G18990) sequence. This sequence was transformed into WT FRI by standard plant transformation with Agrobacterium tumefaciens strain GV3101.

Authentication

The vrn1-4 FRI mutant was backcrossed to WT FRI.
